# Dancing With Parkinson's Disease: The SI-ROBOTICS Study Protocol

**DOI:** 10.3389/fpubh.2021.780098

**Published:** 2021-12-21

**Authors:** Roberta Bevilacqua, Marco Benadduci, Anna Rita Bonfigli, Giovanni Renato Riccardi, Giovanni Melone, Angela La Forgia, Nicola Macchiarulo, Luca Rossetti, Mauro Marzorati, Giovanna Rizzo, Pierpaolo Di Bitonto, Ada Potenza, Laura Fiorini, Federica Gabriella Cortellessa Loizzo, Carlo La Viola, Filippo Cavallo, Alessandro Leone, Gabriele Rescio, Andrea Caroppo, Andrea Manni, Amedeo Cesta, Gabriella Cortellessa, Francesca Fracasso, Andrea Orlandini, Alessandro Umbrico, Lorena Rossi, Elvira Maranesi

**Affiliations:** ^1^Scientific Direction, IRCCS INRCA, Ancona, Italy; ^2^Clinical Unit of Physical Rehabilitation, IRCCS INRCA, Ancona, Italy; ^3^Innovation Lab, Innovation, Marketing and Technology, Exprivia S.p.A., Molfetta, Italy; ^4^Consiglio Nazionale delle Ricerche, Istituto di Tecnologie Biomediche, Milan, Italy; ^5^Grifo Multimedia Srl, Bari, Italy; ^6^Dipartimento Ingegneria Industriale, Università degli Studi di Firenze, Firenze, Italy; ^7^Istituto di BioRobotica, Scuola Superiore Sant'Anna, Pontedera, Italy; ^8^Consiglio Nazionale delle Ricerche, Istituto per la Microelettronica e i Microsistemi, Lecce, Italy; ^9^Consiglio Nazionale delle Ricerche, Istituto di Scienze e Tecnologie della Cognizione, Rome, Italy

**Keywords:** Parkinson's disease, rehabilitation, Irish dance, balance, gait, older people, technology acceptance, social assistive robotics

## Abstract

**Introduction:** Parkinson's disease (PD) is one of the most frequent causes of disability among older people, characterized by motor disorders, rigidity, and balance problems. Recently, dance has started to be considered an effective exercise for people with PD. In particular, Irish dancing, along with tango and different forms of modern dance, may be a valid strategy to motivate people with PD to perform physical activity. The present protocol aims to implement and evaluate a rehabilitation program based on a new system called “SI-ROBOTICS,” composed of multiple technological components, such as a social robotic platform embedded with an artificial vision setting, a dance-based game, environmental and wearable sensors, and an advanced AI reasoner module.

**Methods and Analysis:** For this study, 20 patients with PD will be recruited. Sixteen therapy sessions of 50 min will be conducted (two training sessions per week, for 8 weeks), involving two patients at a time. Evaluation will be primarily focused on the acceptability of the SI-ROBOTICS system. Moreover, the analysis of the impact on the patients' functional status, gait, balance, fear of falling, cardio-respiratory performance, motor symptoms related to PD, and quality of life, will be considered as secondary outcomes. The trial will start in November 2021 and is expected to end by April 2022.

**Discussions:** The study aims to propose and evaluate a new approach in PD rehabilitation, focused on the use of Irish dancing, together with a new technological system focused on helping the patient perform the dance steps and on collecting kinematic and performance parameters used both by the physiotherapist (for the evaluation and planning of the subsequent sessions) and by the system (to outline the levels of difficulty of the exercise).

**Ethics and Dissemination:** The study was approved by the Ethics Committee of the IRCCS INRCA. It was recorded in ClinicalTrials.gov on the number NCT05005208. The study findings will be used for publication in peer-reviewed scientific journals and presentations in scientific meetings.

## Introduction

Parkinson's disease (PD) is one of the most frequent causes of disability among older people. According to the Parkinson's Foundation, more than 10 million people worldwide are living with PD^1^. PD is a chronic-progressive neuro-degenerative disease, characterized by motor disturbances such as bradykinesia (poor and slow movement), tremor at rest, rigidity, posture in flexion and “shuffling gait,” and, not least, a lack of balance resulting in a high risk of falling (1–4). Balance disorders do not always respond to the dopaminergic therapy used in PD. Therefore, physiotherapy becomes an important intervention for their management (5, 6). Postural instability and the resulting falls are among the main factors leading to poorer quality of life, morbidity, and increased mortality risk for people with PD (7). These pathological conditions are routinely treated by rehabilitation approaches aimed at improving static/dynamic balance, recovering walking ability, and preventing falls (3, 8).

Recently, dance has started to be considered as effective training for people with PD, especially for those at the initial stage of PD and with mild/moderate disease severity (9, 10). It has been shown that dance in people with PD can have a positive effect on balance and mobility and can help improving quality of life by reducing symptoms of depression (11–14). Recent studies have shown that sometimes dance can improve balance and functional mobility (15, 16).

Irish dancing, along with tango and other forms of modern dance, may be a valid strategy to motivate people with PD to perform physical activity. Recent studies have shown that Irish dancing can improve balance, mobility, and quality of life through the integration of complex learning patterns of motor skills, dynamic balance, musicality, and socialization (17–20). In addition, Irish music, due to its rhythm, has a predictable pattern that can improve gait and walking (18).

A recent review of the literature (21) pointed out the relevance of different types of dance for improving motor and non-motor symptoms in PD. Despite limited evidence, this review highlights an improvement in motor function, freezing, balance, and gait, as compared to physiotherapy, in people with PD who underwent Irish dance intervention.

In a recent study (22), the researchers have examined the feasibility of a randomized controlled study design and the benefits of an Irish dancing-based intervention, with 90 patients with idiopathic PD. The participants were randomized to Irish set dance classes or to a usual care group. The dance group attended a 1.5-h dancing class each week, for 10 weeks and undertook a home dance program for 20 min, 3 times per week. The usual care group has followed usual care and daily activities. The results have shown that, in the case of mild to moderately severe PD, the Irish dance is a feasible and enjoyable strategy to improve patients' quality of life.

Besides highlighting the positive impact of dancing, several studies have also tried to assess the impact of robot dancers to support older people (23), especially with PD (24). In comparison to human–human partner dance, robot dance partners may support the collection of objective health parameters during the performance, allowing the customization on the basis of the older people's needs and preferences. Furthermore, robot dance partners potentially complement human–human dance by giving the opportunity of also training alone (25).

Moreover, a recent study (26) has demonstrated the acceptability and feasibility of a dancing intervention such as a robot as a partner. The results of this study underline that the 16 older dancers perceived the robot as useful, easy to use, and fun, suggesting a positive attitude toward the system and thus the intervention, evaluated through the Technology Acceptance Model (TAM) scale (27), a specific tool to assess and predict user acceptance of information systems and information technology. The authors have highlighted the need of increasing the complexity of dance exercises with the robot, to favor the engagement of the older dancers.

In line with the literature in the field, the main objective of this paper is to present the protocol for the “SI-ROBOTICS” intervention, aimed at assessing the acceptability of an innovative robotics-based system for engaging patients with Parkinson's disease in an Irish set dancing intervention.

## Methods and Analysis

### Trial Design

The study is designed as a technical feasibility pilot to test the SI-ROBOTICS system on a group of 20 older people with PD at an early stage (Hoen and Yahr Scale 1–2), during an Irish set dancing training. Assessment will be performed at the baseline (T0), after 4 weeks (mid-T1), and after 8 weeks of intervention (end-T2).

The primary aim is to evaluate the acceptability of the SI-ROBOTICS system in a group of patients with PD while performing a rehabilitation treatment based on Irish dancing, using the Unified Theory of Acceptance and Use of Technology (UTAUT) scale (28).

The secondary aim is the analysis of the modification of dimensions related to the general functional status, in terms of gait, balance, fear of falling, cardio-respiratory performance, motor symptoms related to PD, and quality of life.

### Study Setting

This study will be conducted at the Clinical Unit of Physical Rehabilitation of the Istituto Nazionale Ricovero e Cura per Anziani IRCCS INRCA, Ancona, Italy. The last version (second version) of the current protocol is dated May 25, 2021.

### Participants

The inclusion criteria will be:

- Aged 65 and over;
- Capacity to consent;
- Hoen and Yahr scale: 1–2 stage;
- Functional Ambulation Category (FAC) ≥ 2;
- Rankin Scale score ≤ 3;
- Stability of drug treatment for at least 1 month;
- Geriatric Depression Scale 4-items ≤ 1;
- Mini Mental State Examination ≥ 24;
- Maintaining an upright posture ≥ 30, evaluated by a trained physiotherapist during the recruitment.

The exclusion criteria will be:

- Failure to meet the inclusion criteria;
- Concomitant participation in other studies;
- Lack of written informed consent;
- History of syncopal episodes, epilepsy, and vertigo not controlled pharmacologically;
- Serious dysfunction of the autonomic system;
- Severe behavioral syndromes not compensated by drugs;
- Concurrent neurological and/or cardiac diseases;
- Recent femur fracture;
- Chronic medium to severe pain affecting standing or walking.

### Recruitment

Patients will be selected by the outpatient department at the Clinical Unit of Physical Rehabilitation, of IRCCS INRCA, in the Ancona branch. These patients will be contacted to schedule a visit with the clinical team. Once the compliance with the inclusion and exclusion criteria of the study will be verified and the informed consent will be obtained, the doctor will proceed with the baseline evaluation and with the acquisition of gait assessment parameters through G-walk sensor. The trial will start in November 2021 and is expected to end in April 2022.

### Intervention

For this study, 20 patients with PD will be enrolled. Sixteen therapy sessions of 50 min will be conducted (two training sessions per week, for 8 weeks), involving two patients at a time. Cardiac and respiratory activity monitoring will be conducted during dancing treatments in order to detect heart rate and breathing frequency. Participants will have to complete at least 80% of the sessions.

Each session will involve the following activities:

- breathing, relaxation, and postural harmonization exercises (5 min);
- active mobility and stretching exercises (5 min);
- Irish dancing with the SI-ROBOTICS system (35 min);
- relaxation exercises (5 min).

### Platform Description

The SI-ROBOTICS system is composed of multiple technological components. Usability and acceptability of the single components have been evaluated singularly before the system integration. These components are:

- **A robotic platform:** a social robot that will motivate the participants during the sessions, equipped with the Inter Real Sense camera, able to collect information on kinematic parameters such as position of pressure center in relation to the support base, steps, and body symmetry.
- **The let's dance game**: a serious game based on the choreography and a personalized avatar which will guide the participants to perform the dance sessions.
- **Wearable wellness system sensorized shirt:** to collect data on the patient's main clinical parameters (e.g., heart rate, breathing frequency, etc.) during the performance. These data will allow to collect the patient's performance data during the execution of tasks.

In addition, the system is constituted by:

- **The AI reasoner module**: the backend intelligent part of the SI-ROBOTICS system that will allow the adaptation of the dance sessions, based on the users' performance and the data collected through the sensors. In this way, it will be possible to dynamically customize the session according to the user's needs and abilities.
- **The artificial vision setting**: a commercial camera called Inter Real Sense, installed in the experimental setting for the extraction of kinematic parameters. To extract features of interest from the signals, proprietary skeleton tracking software of the Real Sense camera will be used, together with specially developed algorithms for feature extraction.

**Figure 1:**
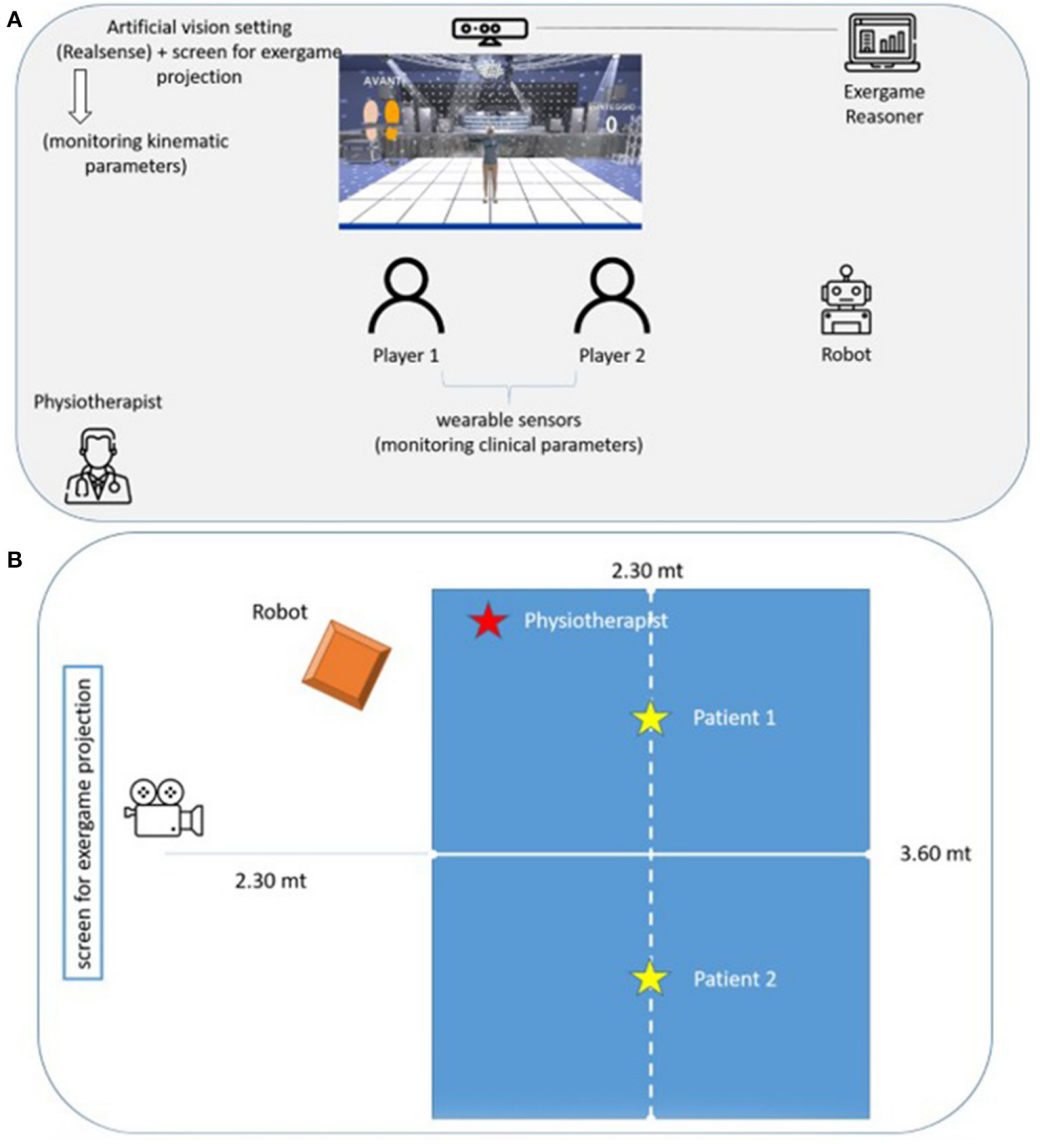
SI-ROBOTIC system **(A)**. Expermental setting **(B)**.

Figures 1A,B shows the experimental setting in a protected environment (gymnasium) and the positioning of the technologies, the user and the physiotherapist, during the training sessions. In particular, the two participants will be placed in front of the screen and the central camera at a distance of 3.40 m. The robot and the physiotherapist will be positioned laterally, at a distance of 3 m. The robot will move without entering the blue area drawn in Figure 1B, using the navigation algorithm based on literature (29, 30).

The technical components are described in details in the following paragraphs.

#### Robotic Platform

**Figure 2:**
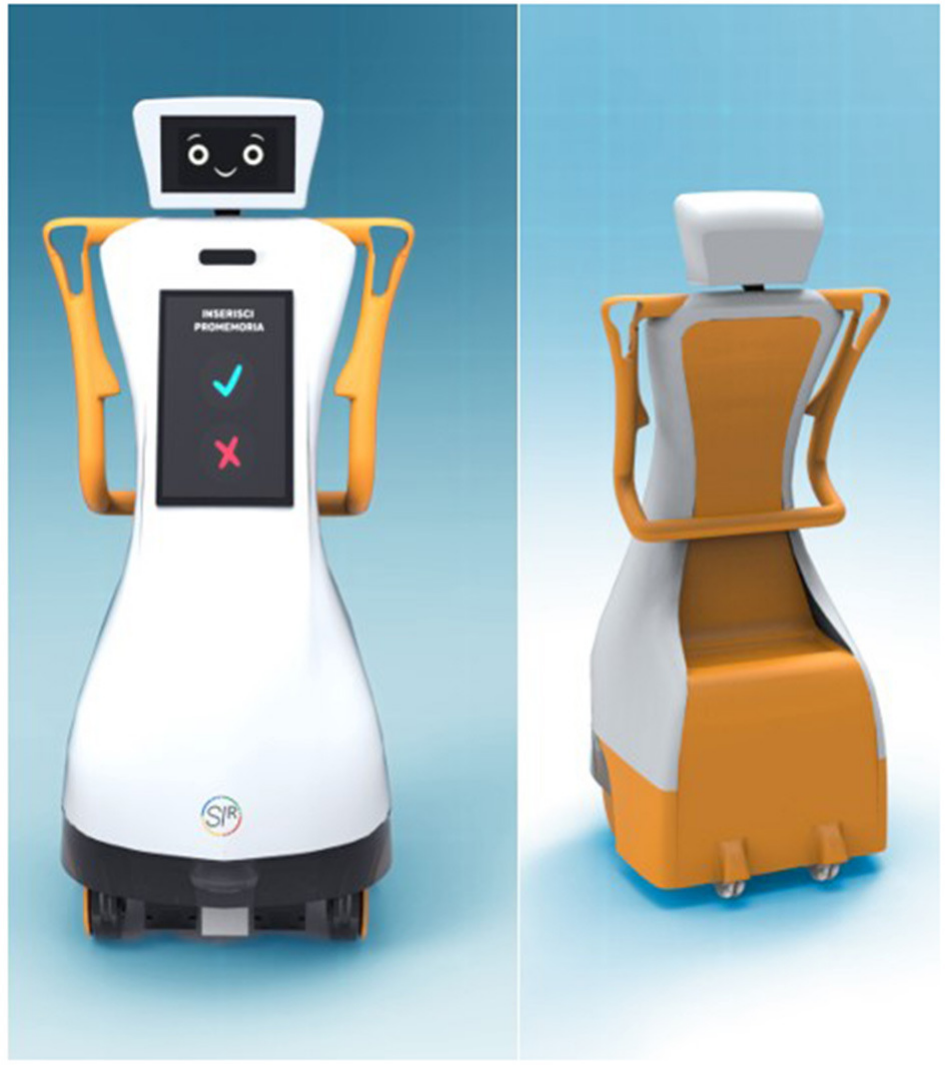
SI-ROBOTICS platform.

From a technical point of view, the SI-ROBOTICS robot is composed of the MoVeR1 robotic platform (Co-Robotics, Italy) as shown in Figure 2, a two-axle autonomous vehicle with two front driving wheels and two rear omni-drive wheels, and an external cover, whose customized design was realized in collaboration with the Department of Design of the University of Genova.

The platform has been equipped with the following commercial sensors to be compliant with the scenario requirement:

- a microphone and a speaker to interact with the participants;
- a tablet, providing a web interface to support the interaction during the service;
- an Intel RealSense D435i RGB-D camera (Intel, USA), which helps the patient–robot interaction, measuring movements, and which performs the remote plethysmography service, by real-time measurement of heart rate and breath rate (31, 32);
- a SICK TIM781 laser 2D, able to detect obstacles and people in the environment.

The robot software is based on the Robot Operating System (ROS) (33), which performs middleware function, i.e., connection layer of all software components strictly related to the robot operation. Among the relevant modules built on ROS, the autonomous navigation module is devoted to performing motor control, avoiding obstacles, planning the best paths, and so on. Furthermore, for the user interaction part, basic modules deal with synthesizing text into dialogs. Other two modules have been developed to link the robot tasks to the application scenarios defined in the project: (1) the social navigation module (34), which is used to make the robot autonomously approach the users, following methodologies in line with social behaviors and (2) the dialogue module, providing motivational statements based on the context and data collected during the dance session, which will be sent to the underlying speech synthesis module for vocal interaction.

#### Let's Dance Game

Let's dance is a game based on Irish dancing sessions. It has been designed to be intuitive and easy to use for both the patient and the physical therapist. After selecting a difficulty level, players are presented as dancers on the screen, along with footprints that suggest the movement to be done.

Each task can vary from simple aerobic exercises (side steps, arm raising, hand-clapping, etc.) to choreographies.

Preliminarily to the use of the game, warm-up exercises will be proposed, in order to minimize physical problems related to the execution of dance steps. After the warm-up, the therapist will log in, will select the involved patients, and will choose the first song and level to be performed. At the end of the dance session, cool-down exercises will be carried out in order to restore the resting condition.

The complexity of the step sequence may increase, ranging from simple steps interspersed with pauses, to sequences of steps, up to complete choreographies, based on the dance movements in the Irish style and Irish music.

An example of steps that will be used: rest, step forward right or left foot, step backward right or left foot, side-step right or left, fake step forward right or left foot, fake step backward right or left foot, full lift right or left arm, partial lift right or left arm.

#### Wearable Wellness System Sensorized Shirt

During the rehabilitation session, patients will wear the Wearable Wellness System (WWS) sensorized shirt, produced by Smartex (http://www.smartex.it/it/prodotti/204-wws). WWS offers a high level of comfort that makes it particularly suitable for monitoring physiological parameters even for long periods. The sensorized garment is produced using natural and synthetic yarns and it can be used as underwear.

The shirt is a fully integrated wearable system for the continuous monitoring of physiological parameters, composed of a sensorized undershirt and an electronic board allowing the continuous monitoring of some clinical parameters relevant to the evaluation of the user's stress and physical state. The shirt is composed of:

- a sensorized garment (t-shirt, in different sizes and profiled for female and male silhouette);
- an electronic device designed for data acquisition, pre-processing, storage, and transmission;
- a customized software for the configuration of the device and the processing, management, transmission of the acquired data.

The system is capable of simultaneously acquiring the following signals:

- ECG signal and
- respiratory signal.

The ECG and respiratory signals are obtained through the use of textile-based fiber sensors integrated directly into the structure of the garment.

The processor on board the electronic device elaborates the acquired signals, through sophisticated signal processing algorithms for artifacts/noise reduction and for the real-time extraction of heart and breathing rate values in a reliable way.

The system has been tested to evaluate its duration in number of washes and times of use. The first signs of aging appear after 50 cycles of delicate washing.

#### AI Reasoner

Its role is to realize the reasoning capabilities necessary to contextualize and adapt the behavior of the SI-ROBOTICS system according to the clinical objectives of the session and the “dynamic performance” of the users. During the session, the artificial vision system and the installed physiological sensors will produce a flow of data used by the reasoner to monitor the progress of the session and the status of the users.

The step sequences generated by the reasoner will be sent to the game for the dance session execution. During the exercise, the reasoner's role will be to monitor performance and to dynamically adapt the exercise when necessary.

Generally, there are two types of scenarios that we will consider during the course of the session:

- Complete session: in this scenario, the reasoner will not face any special situations and/or conditions during the exercise.
- Interruption or dynamic adaptation of the session: in this scenario, the reasoner encounters abnormal situations that require direct intervention of the system and dynamic adaptation of the session.

Moreover, the reasoner will suggest to the therapist the change from one level of difficulty to another during the treatment, on the basis of variations calculated on patient-specific baseline values taken at the beginning of the intervention.

#### Artificial Vision Setting

As already said, another Intel RealSense depth camera will be installed in the experimental setup and will be used for the extraction of kinematic parameters, thanks to the Skeleton Tracking SDK developed by Cubemos (München, Germany). Indeed, for each detected skeleton, the spatial coordinates (x, y, and z) and the detection confidence of 18 joints will be estimated. For each step involved in the Irish dance subsession, the following clinical key performance indicators (KPIs), agreed with the physiotherapists, will be extracted in real-time:

- step symmetry;
- symmetry between left and right foot (computed for lower limbs);
- symmetry between left and right arm, with respect to the request, i.e., 90° or 180° lift exercise (computed for upper limbs);
- inclination of the trunk: the flexion of the trunk will be measured during the dance. A warning will be triggered if the person tilts the trunk forward exceeding the tolerance.
- the center of gravity: the stability of the subject will be evaluated by calculating the position of the center of gravity. The system will give a warning when the projection of the center of mass exits the support base defined by the length and position of the feet.

#### A Middleware to Facilitate Module Interaction

The interoperable platform is intended as a distributed middleware, i.e., a set of protocols, interfaces, and services for the development of applications capable of processing large amounts and streams of data from heterogeneous sources.

For the rehabilitation program, the platform will monitor the values and allow the exchange, and processing of data through artificial intelligence algorithms, thus obtaining information useful to the physical therapist in the evaluation of the patient's performance.

This is possible through the main components internal to the middleware:

- Event bus interoperability framework: it allows communication and data exchange between the components through the use of *connectors* and *topics* according to the *publish–subscribe* mechanism (a publisher publishes an event to the Event Bus; on the other hand, the subscriber receives this event).
- Stream and batch processing for the data analysis in real-time or batch.
- Persistence layer: it allows the persistence of circulating information.

### Outcomes

All outcomes will be measured following a standardized operating procedure. Table 1 shows the primary and secondary outcomes.

**Table 1:** Outcomes and clinical assessments.

**Outcome(s)**	**Clinical assessment**
Primary: acceptability of the SI-ROBOTICS system	UTAUT
Secondary: gait improvement (increase in walking speed)	Gait speed during 6 MWT though (G-Walk sensor)
Secondary: balance	POMA
Secondary: fear of falling	FES-I Short form
Secondary: general functional status	SPPB and TUG
Secondary: user satisfaction	GAS
Secondary: cardiac and respiratory performance	HR and RR using wearable sensors
Secondary: quality of life	SF-12

*UTAUT, Unified Theory of Acceptance and Use of Technology; 6 MWT,6 Minute Walking Test; POMA, Performance-Oriented Mobility Assessment; FES-I, Short Falls Efficacy Scale – International; SPPB, Short Physical Performance Battery; TUG, Time Up and Go; GAS, Goal Attainment scale; HR, Heart Rate; RR, Respiratory Rate; SF-12, Short Form (12)*.

A summary of all data collected and when these are collected is provided in Table 2.

**Table 2:** Schedule of assessment and outcome measures.

	**R**	**T0**	**T1**	**T2**
Mini Mental State Examination (MMSE)	✓			
Functional Ambulation Category (FAC)	✓			
Rankin Scale	✓			
Hoen and Yahr Scale	✓			
Geriatric Depression Scale−5 items (GDS)	✓			
Socio-demographics check list		✓		
Barthel Index		✓		
Short Form Health Survey (SF-12)		✓		✓
Assistive Device Predisposition Assessment—Scale E (ATDPA)		✓		
Unified Theory of Acceptance and Use of Technology (UTAUT)				✓
Performance-Oriented Mobility Assessment (POMA)		✓	✓	✓
Short Falls Efficacy Scale—International (FES—I)		✓	✓	✓
Short Physical Performance Battery (SPPB)		✓	✓	✓
Unified Parkinson's Disease Rating Scale—III (UPDRS—III)		✓	✓	✓
Timed Up and GO (TUG)		✓	✓	✓
6 Minute walking test (6 MWT)		✓	✓	✓
Goal Attainment scale (GAS)		✓		✓
*Ad hoc* scale on satisfaction with the SI-ROBOTICS intervention				✓

*R, Recruitment; T0, Start of treatment; T1, Mid-treatment (4 weeks); T2, end of treatment (8 weeks). ✓indicates when the test has been performed*.

The scales which will be used during the evaluations are described below.

#### Mini-Mental State Examination

Mini-Mental State Examination was designed as a clinical method for grading cognitive impairment. The score ranges from 0 to 30: scores ≥ 24 indicate normality between 18 and 23 indicate mild cognitive impairment, between 11 and 17 average cognitive deficits, scores ≤ 10 severe cognitive impairments. The reported score is corrected according to age and education (35).

#### Functional Ambulation Categories

The scale is used to classify the severity level of gait disturbances in neurological disorders. It provides a hierarchical classification from level 0 (impossible walking) to level 5 (no limitation) (36).

#### Rankin Scale

It is a simple scale for the evaluation of the outcomes following a stroke. Reliability is well-defined. The individual categories are essentially based on patient mobility. There are 6 grades of classification from 0 to 5, where 0 means independence and 5 means severe disability (37).

#### Hoehn and Yahr Scale

This scale is used in the medical field to describe the symptoms of PD progression. It was originally published in 1967 in the Neurology Journal by Melvin Yahr and Margaret Hoehn, and included stages 1–5. Since then, a modified scale has been proposed, with the addition of stages 1.5 and 2.5 describing the intermediate course of the disease (38).

#### Geriatric Depression Scale 5-Items Version

This questionnaire assesses the current condition of the patient's mood. For the screening required by our study, only the first five items of the scale can be used. The answers highlighted indicate the statements expected by a non-depressed subject (39).

#### Barthel Index

Barthel index is an ordinal scale used to measure a subject's performance in everyday life activities. The index analyzes ten variables that describe the activities of daily life and mobility. A high overall score is associated with a greater probability of being able to live at home independently after discharge from the hospital (40).

#### SF-12 Health Survey

The SF-12 is composed of 12 items that produce two measurements related to two different aspects of health: physical health and mental health. The subject is asked to answer on how he feels and how he is able to carry out the usual activities, evaluating the current day and the four previous weeks (41).

#### Assistive Device Predisposition Assessment

The purpose of the tool is to assess the user expectations about technological devices (42).

#### Unified Theory of Acceptance and Use of Technology

The UTAUT (28) scale is made up of four constructs (performance expectancy, PE; effort expectancy, EE; social influence, SI; facilitating conditions, FC) that determine the level of acceptance of a technology by users, in terms of both attitudes toward the system (behavioral intention, BI) and its use (use behavior, USE). In addition, UTAUT incorporates four moderators (gender, age, experience, and voluntariness) that regulate the existing relationships between independent quantities (constructs) (PE, EE, SI, and FC) and dependent quantities (constructs) (BI, USE).

#### Tinetti's Scale or Performance-Oriented Mobility Assessment

Tinetti's scale is a tool used to evaluate balance and gait performance. The test is clinically used to determine the mobility status of a subject or to assess changes in balance and gait time. The total POMA (POMA-T) consists of two sub-scales: the balance evaluation scale (“balance scale” or POMA-B) and the gait evaluation scale (“gait scale” or POMA-G) (43).

#### Short Falls Efficacy Scale – International

The scale measures the “fear of falling.” The cut-offs for the fear of falling are divided as follows: a score between 7 and 8 indicates a low concern, between 9 and 12 a moderate concern, and between 14 and 28 a high concern (44).

#### Short Physical Performance Battery

The SPPB scale is a short battery of tests designed to assess the function of the lower limbs. This scale consists of three different sections: balance assessment, evaluation of walking on four linear meters, evaluation of the ability to perform, for five consecutive times, the sit to stand from a chair, without using the upper limbs. The total scale score, therefore, has a range from 0 to 12 (45).

#### Unified Parkinson's Disease Rating Scale – III

The UPDRS is the most widely used rating scale in assessing the prognosis of Parkinson's disease. The total number of items investigated in this clinical assessment is 65, which can be scored from 0 to 4. A score of 0 is given in a situation of normality with respect to the problem being investigated, while if there is a minimal impairment, 1 point is given, if it is mild it is indicated by 2 points, while if it is moderate it is marked with a 3, in a more serious situation a score of 4 is given (46).

#### Timed Up and GO

TUG is a simple test to measure a person's level of mobility and requires static and dynamic balancing skills. It measures the time and takes a person to get up from a chair, walk three meters, turn around, return to the chair and sit down again (47).

#### Six Minute Walking Test

6MWT is a test that allows measurement of a patient's residual functional capacity. The test is performed by asking the patient to walk for 6 min along a corridor with a rigid walking surface. The 6MWT is based on a so-called self-pace mode, i.e., the patient chooses the intensity of effort (48).

#### Goal Attainment Scale

GAS is a measure referring to an individualized criterion for assessing the achievement of objectives. Five possible levels are defined for each objective: −2 result much lower than expected; −1 result lower than expected; 0 achievement; +1 result higher than expected; +2 result much higher than expected. Behavioral objectives are usually measured dichotomously: as YES or NO, “achieved” or “not achieved” (49).

*Ad hoc scale on satisfaction with the SI-ROBOTICS intervention:* The scale consists of 47 items, the domains of which are:

- Social Presence: its sub-dimensions are overall perceived social presence, psycho-behavioral interaction, and trust. The overall perceived social presence consists of six items. Subjects are asked to rate their opinion on a five-point scale. The psycho-behavioral interaction scale consists of five items. The trust scale consists of four items.
- HRI: its sub-dimensions are Language, Communication, and Perceived safety during movement. The language scale is composed of two items, built *ad-hoc* to verify the speech capability of the robotic platform. Communication is composed of three *ad-hoc* developed questions. Finally, the perceived safety scale is built on four items. Two of them are built *ad-hoc* for the project, while the other two are an adapted version of the standardized Godspeed tool on perceived safety, where users are asked to rate their emotional state in relation to the speed of the robot.
- Usability: its sub-dimensions are perceived as easy to use (four items), perceived usefulness (six items), and satisfaction with use (three items). All questions were constructed *ad-hoc* for the SI-ROBOTICS system.
- Acceptability: its sub-dimensions are anxiety (three items), attitude (three items), perceived adaptability (four items), and intention to use (four items). All questions were constructed *ad-hoc* for the SI-ROBOTICS system.

#### Instrumental Gait Analysis

Gait analysis will be performed through the G-Walk (BTS Bioengineering) during the 6MWT. G-Sensor is a wearable system for gait and movement assessment. It allows having a kinematic evaluation of the trunk and of the spatio-temporal parameters recorded during the different tests and integrated protocols.

### Risk Management and Mitigation

The risk of falling is major during any rehabilitation program. The SI-ROBOTICS platform is not only designed to partner dance with patients with PD, but also to motivate them during the training and to capture information on kinematics at a secure distance. Moreover, the robotic platform is equipped with obstacles avoidance sensors at the basis to counteract the risk of hurting the participants during the performance. However, a dedicated physiotherapist will be present and will supervise the entire session, in order to intervene promptly in case of fall risk or any other emergency.

In case a fall occurs, the physiotherapist will follow this procedure: they will ask the patient to stay still before checking for pain, loss of sensation (feeling), and loss of movement in arms and/or legs which might indicate a fracture. If there is no evident injury and no signs of a change in health, in line with the person's wishes they allow them to get up independently if possible, or assist the patient in doing so. However, any adverse event related to the training will be covered by the Institution's insurance.

### Data Management

The project committed to the maintenance of participants' anonymity and confidentiality throughout all procedures, such as screening, recruitment, testing, evaluation, and dissemination procedures. Data collection, usage, and storage procedures complied with national laws and the EU's General Data Protection Regulation (GDPR) such as the commitment of participants' the right to access, right to be informed, right to withdraw, and right to data erasure. Data collection will be compliant with the principle of data minimization, i.e., the collection of personal information from study participants will be limited to what is directly relevant and necessary to accomplish the specific goals of the testing and evaluation work packages. Data entry will be carried out using specific software, providing blocks, and data entry checks, in order to reduce the number of entry errors. All screening data were will be discarded upon the project completion. During the testing procedures, all visual, auditory, and sensory data that the robot collects and processes in order to function as planned will be discarded after the procedures have been completed. The exception to this is the collection of the number of interactions that the robot logs with each participant. However, these interactions will be anonymous. All research data shall be made openly available for secondary analysis 3 years after the project completion.

### Data Analysis

The first step of the data analysis will deal with the description of the sample. Continuous variables will be reported as either mean and standard deviation or median and interquartile range on the basis of their distribution (assessed using the Kolmogorov–Smirnov test). Categorical variables will be expressed as an absolute number and percentage. The Mann–Whitney *U*-tests (for non-normal distribution), or the chi-square tests (normal or non-normal) will be used to compare the independent and dependent variables between the pre- and post-conditions, in addition to simple descriptive statistics (means, medians, and SDs as appropriate).

In order to verify the achievement of the primary endpoint (i.e., acceptability of the SI-ROBOTICS system), subscales of the UTAUT will be calculated. Means and standard deviation or medians and IQR of the scores will be reported according to their distribution. Correlation coefficients (Pearson for normally distributed variables, Spearman for non-normally distributed variables) of the UTAUT sub-scales with the other rating scales at each stage of the study and with the main characteristics of the subjects will be calculated to check for potential determinants of higher acceptability.

## Discussion

In this study, we have described an intervention protocol to evaluate the acceptability of the SI-ROBOTICS system in a group of patients with PD, while performing a rehabilitation treatment based on Irish dancing.

While the focus of common rehabilitative approaches for patients with PD relies on the improvement of static and dynamic balance, walking, and falls (50–52). It was recently found that performance improvements after technology-delivered balance training correlate with evident neurobiological changes in the cerebral cortex. In particular, combining the high personalization and flexibility of the technological system (53) with smart objects or robots to encourage the participants during the training with Irish dancing (54–58), will strongly motivate the patients with PD in participating in the rehabilitation program, by promoting satisfactory pattern (59) and distracting by fatigue (60).

Moreover, the SI-ROBOTICS intervention has the potential to reduce several barriers to exercise, such as lack of self-confidence, lack of skills, lack of support, costs, and lack of physical activity options (61), while enhancing motivators based on the users' profile. It will also provide evidence to private and public health facilities of an innovative therapeutic treatment based on dance, making the physical therapy more similar to a leisure activity, while improving compliance to an effective treatment, and thus counteracting the exacerbation of motor and non-motor symptoms.

However, there are also limitations in the applicability of the intervention. First of all, at present, participation is limited to patients with PD at an initial stage of the disease. In the future, it is planned to arrange a larger study to better understand the opportunity of involving more participants at different stages of the disease. Moreover, participants need to be controlled in regards to adherence to medical treatments, to avoid the risk of bias during the performance.

## Ethics and Dissemination

### Ethics and Confidentiality

This study was approved by the Ethic Committee of the Istituto Nazionale Ricovero e Cura per Anziani, (IRCCS INRCA) on the June 17, 2021. It was recorded in ClinicalTrials.gov on August 13, 2021 with the number NCT05005208. Any protocol modifications will be notified to the above-mentioned Ethics Committee. The same committee is in charge of data monitoring and periodically assesses the progress of the protocol and compliance with what was declared. The principles of the Declaration of Helsinki and Good Clinical Practice guidelines will be adhered to. Participants in this study provided written informed consent.

Personal data collected during the trial will be handled and stored in accordance with the General Data Protection Regulation (GDPR) 2018. The use of the study data will be controlled by the principal investigator. All data and documentation related to the trial will be stored in accordance with applicable regulatory requirements and access to data will be restricted to authorized trial personnel.

### Dissemination of Research Findings

The study findings will be used for publication in peer-reviewed scientific journals and presentations in scientific meetings. Summaries of the results will also be made available to investigators for dissemination within their clinics.

## Ethics Statement

The studies involving human participants were reviewed and approved by IRCCS INRCA Ethical Committee. The patients/participants provided their written informed consent to participate in this study.

## Author Contributions

EM and RB led the design and writing of the pilot RCT protocol. GRR, AB, and LRossi helped with the development of the participant identification plan and provided advice on other key study issues. MB helped with the design of the intervention. EM, RB, FF, and GC lead the collection, management, and statistical analysis of the data. GM, AL, NM, and LRosse contributed to the middleware section description and development. MM and GRi contributed to the analysis of the physiological parameters section description. LF, CL, FC, and FGC contributed to the robotic platform section. AL, GRe, ACa, and AM contributed to wearable sensors section description and development. FF, GC, AU, AO, and ACe contributed to the reasoner section description and development. PD and AP contributed to the Let's dance game section description and development. All authors contributed important intellectual content to the written protocol and approved the final version for publication.

## Funding

The study has received funding under the project SI-ROBORICS Healthy and active aging through SocIal ROBOTICS (ARS01_01120), funded by the Italian Ministry of Education, Universities, and Research, under the National Operational Program Area Technologies for Living environments.

## Conflict of Interest

GM, AF, NM, and LRosse, were employed by company Exprivia S.p.A. PD and AP were employed by company Grifo Multimedia Srl. The remaining authors declare that the research was conducted in the absence of any commercial or financial relationships that could be construed as a potential conflict of interest.

## Publisher's Note

All claims expressed in this article are solely those of the authors and do not necessarily represent those of their affiliated organizations, or those of the publisher, the editors and the reviewers. Any product that may be evaluated in this article, or claim that may be made by its manufacturer, is not guaranteed or endorsed by the publisher.

## Footnotes

^1^Available online at: https://www.parkinson.org/Understanding-Parkinsons/Statistics.

## References

[1] de Rijk MC Breteler MMB Graveland GA Ott A Grobbee DE van der Meche FGA . Prevalence of Parkinson's disease in the elderly. Rotterdam Study. (1995) 45. 10.1212/WNL.45.12.2143 8848182

[2] Allen NE Sherrington C Paul SS Canning CG. Balance and falls in Parkinson's disease: a meta-analysis of the effect of exercise and motor training. Mov Disord. (2011) 26:1605–15. 10.1002/mds.23790 21674624

[3] Mansfield A Wong JS Bryce J Knorr S Patterson KK. Does perturbation-based balance training prevent falls? Systematic review and meta-analysis of preliminary randomized controlled trials. Phys Ther. (2015) 95:700–9. 10.2522/ptj.20140090 25524873

[4] Draganski B Gaser C Busch V Schuierer G Bogdahn U May . Neuroplasticity: changes in grey matter induced by training. Nature. (2004) 427:311–12. 10.1038/427311a 14737157

[5] Park JH Kang YJ Horak FB. What is wrong with balance in Parkinson's disease? J Mov Disord. (2015) 8:109–14. 10.14802/jmd.15018 26413237 PMC4572660

[6] Ellis T Goede CJ Feldman R Wolters EC Kwakkel G Wageenar RC. Efficacy of a physical therapy program in patients with Parkinson's disease: a randomized clinical trial. Arch Phys Med Rehabil. (2005) 4:626–32. 10.1016/j.apmr.2004.08.008 15827910

[7] Palakurthi B Burugupally SP. Postural instability in Parkinson's disease: a review. Brain Sci. (2019) 9:239. 10.3390/brainsci9090239 31540441 PMC6770017

[8] Gordt K Gerhardy T Najafi B Schwenk M. Effects of wearable sensor-based balance and gait training on balance, gait, and functional performance in healthy and patient populations: a systematic review and meta-analysis of randomized controlled trials. Gerontology. (2018) 64:74–89. 10.1159/000481454 29130977

[9] Earhart GM. Dance as therapy for individuals with Parkinson's disease. Eur J Phys Rehabil Med. (2009) 45:231–8. 19532110 PMC2780534

[10] Emmanouilidis S Hackney ME Slade SC Heng H Jazayeri D Morris ME. Dance is an accessible physical activity for people with Parkinson's Disease. Parkinson's Disease. (2018) 2021:7516504. 10.1155/2021/7516504 34721836 PMC8556098

[11] Duncan RP Earhart GM. Randomized controlled trial of communitybased dancing to modify disease progression in Parkinson disease. Neurorehabil Neural Repair. (2012) 26:132–43. 10.1177/1545968311421614 21959675

[12] Hackney ME Earhart GM. Health-related quality of life and alternative forms of exercise in Parkinson disease. Parkinsonism Relat Disord. (2009) 15:644–8. 10.1016/j.parkreldis.2009.03.003 19329350 PMC2783812

[13] Kiepe MS Stockigt B Keil T. Effects of dance therapy and ballroom dances on physical and mental illness: a systematic review. Arts Psychother. (2012) 39:404–11. 10.1016/j.aip.2012.06.001

[14] Soh ES Morris ME McGinley JL. Determinants of health-related quality of life in Parkinson's disease: a systematic review. Parkinsonism Relat Disord. (2011) 17:1–9. 10.1016/j.parkreldis.2010.08.012 20833572

[15] Hackney ME Kantorovich S Levin R Earhart GM. Effects of tango on functional mobility in Parkinson's disease: a preliminary study. J Neurol Phys Ther. (2007) 31:173–9. 10.1097/NPT.0b013e31815ce78b 18172414

[16] Coubard OA Duretz S Lefebvre V Lapalus P Ferrufino L. Practice of contemporary dance improves cognitive flexibility in aging. Front Aging Neurosci. (2011) 3:13. 10.3389/fnagi.2011.00013 21960971 PMC3176453

[17] Shanahan J Morris ME Bhriain ON Volpe D Richardson M Clifford AM. Is Irish set dancing feasible for people with Parkinson's disease in Ireland. Complement Ther Clin Pract. (2015) 21:47–51. 10.1016/j.ctcp.2014.12.002 25557584

[18] Volpe D Signorini M Marchetto A Lynch T Morris ME. A comparison of Irish set dancing and exercises for people with Parkinson's disease: a phase II feasibility study. BMC Geriatr. (2013) 13:1–6. 10.1186/1471-2318-13-54 23731986 PMC3685562

[19] Murphy P. The Flowing Tide. Ireland: Mercier Press (2000).

[20] Foley C. The irish Céilí: a site for constructing, experiencing, and negotiating a sense of community and identity. Dance Res. (2011) 29:43–60. 10.3366/drs.2011.0004

[21] Ismail SR Lee SWH Merom D Megat Kamaruddin PSN Chong MS Ong T . Evidence of disease severity, cognitive and physical outcomes of dance interventions for persons with Parkinson's Disease: a systematic review and meta-analysis. BMC Geriatr. (2021) 21:503. 10.1186/s12877-021-02446-w 34551722 PMC8456607

[22] Shanahan J Coman L Ryan F Saunders J O'Sullivan K Ni Bhriain O . To dance or not to dance? A comparison of balance, physical fitness and quality of life in older Irish set dancers and age-matched controls. Public Health. (2016) 141:56–62. 10.1016/j.puhe.2016.07.015 27932016

[23] Kattenstroth JC Kalisch T Holt S Tegenthoff M Dinse HR. Six months of dance intervention enhances postural, sensorimotor, and cognitive performance in elderly without affecting cardio-respiratory functions. Front Aging Neurosci. (2013) 5:5. 10.3389/fnagi.2013.00005 23447455 PMC3581819

[24] Chen TL Bhattacharjee T McKay JL Borinski JE Hackney ME Ting LH . Evaluation by expert dancers of a robot that performs partnered stepping via haptic interaction. PLoS ONE. (2015) 10:e0125179. 10.1371/journal.pone.0125179 25993099 PMC4438977

[25] Hackney M Kantorovich S Earhart G. A study on the effects of argentine tango as a form of partnered dance for those with Parkinson disease and the healthy elderly. Am. J. Dance Ther. (2007) 29:109–27. 10.1007/s10465-007-9039-2

[26] Chen TL Bhattacharjee T Beer JM Ting LH Hackney ME Rogers WA . Older adults' acceptance of a robot for partner dancebased exercise. PLoS ONE. (2017) 12:e0182736. 10.1371/journal.pone.0182736 29045408 PMC5646767

[27] Davis FD. Perceived usefulness, perceived ease of use, and user acceptance of information technology. MIS Q. (1989) 13:319–40. 10.2307/249008

[28] Venkatesh V . User acceptance of information technology: toward a unifed view. MIS Q. (2003) 27:425–78. 10.2307/30036540

[29] Khan F Alakberi A Almaamari S Beig AR. Navigation algorithm for autonomous mobile robots in indoor environments. (2018) 1–6. 10.1109/ICASET.2018.837683427483264

[30] Yasuda Y Martins L Cappabianco F. Autonomous visual navigation for mobile robots: a systematic literature review. ACM Comput Surv. (2020) 53:1–34. 10.1145/3368961

[31] Casalino G Castellano G Pasquadibisceglie V Zaza G. Contact-less real-time monitoring of cardiovascular risk using video imaging and fuzzy inference rules. Information. (2019) 10:9. 10.3390/info10010009

[32] Caroppo A Leone A Manni A Siciliano P. Vision-Based Heart Rate Monitoring in the Smart Living Domains. In Italian Forum of Ambient Assisted Living. Cham: Springer (2021).

[33] Quigley M Conley K Gerke B Faust J Foote T Leibs J . ROS: an open-source Robot Operating System. In: ICRA Workshop on Open Source Software, Vol. 3 (2019).

[34] Sorrentino A Khalid O Coviello L Cavallo F Fiorini L. Modeling human-like robot personalities as a key to foster socially aware navigation. In: 2021 30th IEEE International Conference on Robot and Human Interactive Communication (RO-MAN). IEEE (2021).

[35] Folstein MF Folstein SE McHugh PR. Mini-mental state. A pratical method for grading the cognitive state of patients for the clinician. J Psychiatric Res. (1975) 12:189–98. 10.1016/0022-3956(75)90026-6 1202204

[36] Holden MK Gill KM Magliozzi MR. Gait assesment for neurologically imparired. Standards for outcome assessment. Phys. Ther. (1986) 66:1530–9. 10.1093/ptj/66.10.1530 3763704

[37] Van Swieten JC Koudstaal PJ; Visser MC Schouten HJA van Gijn J. Interobserver agreement for the assessment of handicap in stroke patients. Stroke. (1988) 19:604–07. 10.1161/01.STR.19.5.604 3363593

[38] Hoehn M Yahr M. Parkinsonism: onset, progression and mortality. Neurology. (1967) 17:427–42. 10.1212/WNL.17.5.427 6067254

[39] Rinaldi P Mecocci P Benedetti C Ercolani S Bregnocchi M Menculini G . Validation of the five-item geriatric depression scale in elderly subjects in three different settings. J Am Geriatr Soc. (2003) 51:694–8. 10.1034/j.1600-0579.2003.00216.x 12752847

[40] Mahoney FI Barthel DW. Functional evaluation: the Barthel Index. Md State Med J. (1965) 14:61–5. 10.1037/t02366-000 14258950

[41] Ware J Kosinski M Keller SD. A 12-Item Short-Form Health Survey: construction of scales and preliminary tests of reliability and validity. Med Care. (1996) 3:220–33. 10.1097/00005650-199603000-00003 8628042

[42] Scherer MJ Cushman LA. Measuring subjective quality of life following spinal cord injury: a validation study of the assistive technology device predisposition assessment. Disabil Rehabil. (2001) 23:387–93. 10.1080/09638280010006665 11394589

[43] Tinetti ME. Performance-oriented assessment of mobility problems in elderly patients. J Am Geriatr Soc. (1986) 34:119–26. 10.1111/j.1532-5415.1986.tb05480.x 3944402

[44] Ruggiero C Mariani T Gugliotta R Gasperini B Patacchini F Nguyen HN . Validation of the Italian version of the falls efficacy scale international (FES-I) and the SHORT FES-I in community dwelling older persons. Arch Gerontol Geriatr. (2009) 49 (Suppl.):211–9. 10.1016/j.archger.2009.09.031 19836635

[45] Guralnik JM Simonsick EM Ferrucci L Glynn RJ Berkman LF Blazer DG . A short physical performance battery assessing lower extremity function: association with self-reported disability and prediction of mortality and nursing home admission. J Gerontol. (1994) 49:M85–94. 10.1093/geronj/49.2.M85 8126356

[46] Goetz CG Fahn S Martinez-Martin P Poewe W Sampaio C Stebbins GT . Movement Disorder Society-sponsored revision of the unified Parkinson's disease rating scale (MDS-UPDRS): process, format, and clinimetric testing plan. Mov Disord. (2007) 22:41–7. 10.1002/mds.21198 17115387

[47] Podsiadlo D Richardson S. The timed “up & go”: a test of basic functional mobility for frail elderly persons. J Am Geriatr Soc. (1991) 39:142–8. 10.1111/j.1532-5415.1991.tb01616.x 1991946

[48] Balke B. A Simple Field Test for the Assessment of Physical Fitness. rep 63-6. Rep. Civ. Aeromed Res. Inst. (1963). p. 1–8. Available online at: https://www.faa.gov/data_research/research/med_humanfacs/oamtechreports/1960s/media/am63-06.pdf 14131272

[49] KJ Ottenbacher A Cusick. Goal attainment scaling as a method of clinical service evaluation. Am J Occup Ther. (1990) 44:519–25. 10.5014/ajot.44.6.519 2353720

[50] Cakit BD Saracoglu M Genc H Erdem HR Inan L. The effects of incremental speed-dependent treadmill training on postural instability and fear of falling in Parkinson's disease. Clin Rehabil. (2007) 21:698–705. 10.1177/0269215507077269 17846069

[51] Maranesi E Riccardi GR Lattanzio F Di Rosa M Luzi R Casoni E . Randomised controlled trial assessing the effect of a technology-assisted gait and balance training on mobility in older people after hip fracture: Study protocol. BMJ Open. (2020) 10:e035508. 10.1136/bmjopen-2019-035508 32546491 PMC7299027

[52] Bevilacqua R Casaccia S Cortellessa G Astell A Lattanzio F Corsonello A . Coaching through technology: a systematic review into efficacy and effectiveness for the ageing population. Int J Environ Res Public Health. (2020) 17:1–14. 10.3390/ijerph17165930 32824169 PMC7459778

[53] Bevilacqua R Maranesi E Riccardi GR Di Donna V Pelliccioni P Luzi R . Non-immersive virtual reality for rehabilitation of the older people: a systematic review into efficacy and effectiveness. J Clin Med. (2019) 8:1882. 10.3390/jcm8111882 31694337 PMC6912349

[54] Papetti A Iualé M Ceccacci S Bevilacqua R Germani M Mengoni M. Smart objects: an evaluation of the present state based on user needs. In: Lecture Notes in Computer Science (including subseries Lecture Notes in Artificial Intelligence and Lecture Notes in Bioinformatics) Volume 8530 LNCS, Pages 359 - 3682014 2nd International Conference on Distributed, Ambient and Pervasive Interactions, DAPI 2014 - Held as Part of 16th International Conference on Human-Computer Interaction, HCI International 2014. Heraklion (2014). 105287 p.

[55] Casaccia S Revel GM Scalise L Bevilacqua R Rossi L Paauwe RA . Social robot and sensor network in support of activity of daily living for people with dementia. In: Communications in Computer and Information Science Volume 1117, Pages 128 - 1352019 4th Dementia Lab Conference, D-Lab 2019. Eindhoven (2019). 233299 p.

[56] Bevilacqua R Felici E Marcellini F Glende S Klemcke S Conrad I . Robot-era project: Preliminary results on the system usability. In: Lecture Notes in Computer Science (including subseries Lecture Notes in Artificial Intelligence and Lecture Notes in Bioinformatics) Open AccessVolume 9188, Pages 553 - 5612015 4th International Conference on Design, User Experience and Usability, DUXU 2015 Held as Part of 17th International Conference on Human-Computer Interaction, HCI International 2015. Los Angeles, CA (2015). 123219 p.

[57] Cavallo F Esposito R Limosani R Manzi A Bevilacqua R Felici E . Robotic services acceptance in smart environments with older adults: user satisfaction and acceptability study. J Med Int Res. (2018) 20:e264. 10.2196/jmir.9460 30249588 PMC6231879

[58] Bevilacqua R Felici E. Cavallo F Amabili G Maranesi E. Designing acceptable robots for assisting older adults: a pilot study on the willingness to interact. Int J Environ Res Public Health. (2021) 18:10686. 10.3390/ijerph182010686 34682433 PMC8536134

[59] Blood AJ Zatorre RJ. Intensely pleasurable responses to music correlate with activity in brain regions implicated in reward and emotion. Proc Natl Acad Sci USA. (2001) 98:11818–23. 10.1073/pnas.191355898 11573015 PMC58814

[60] Lim HA Miller K Fabian C. The effects of therapeutic instrumental music performance on endurance level, self-perceived fatigue level, and selfperceived exertion of inpatients in physical rehabilitation. J Music Ther. (2011) 48:124–48. 10.1093/jmt/48.2.124 21938889

[61] van Schijndel-Speet M Evenhuis HM van Wijck R van Empelen P Echteld MA. Facilitators and barriers to physical activity as perceived by older adults with intellectual disability. Mental Retardation. (2014) 52:175–86. 10.1352/1934-9556-52.3.175 24937743

